# Phylo-Epigenetic Conservation and CpG Erosion in *OCT4*, *SOX2*, and *hTERT* Intragenic CpG Islands: A Waddingtonian Perspective on Mammalian Developmental Evolution

**DOI:** 10.3390/genes16091102

**Published:** 2025-09-18

**Authors:** Simeon Santourlidis

**Affiliations:** Epigenetics Laboratory for Human Health and Longevity, Institute of Transplantation Diagnostics and Cell Therapeutics, Medical Faculty, Heinrich-Heine University Duesseldorf, Moorenstr. 5, 40225 Duesseldorf, Germany; simeon.santourlidis@med.uni-duesseldorf.de

**Keywords:** CpG island, epigenetics, DNA methylation, primates, *OCT4*, *SOX2*, *hTERT*, phylo-epigenetics, comparative genomics, developmental regulation

## Abstract

**Background/Objectives:** Developmental biologist Conrad Waddington proposed that evolution is shaped not only by genetic mutations and natural selection but also by environmentally responsive developmental mechanisms. Building on this premise, the epigenetic regulation of three master genes central to mammalian embryogenesis—*OCT4*, *SOX2*, and *hTERT*—focusing on their intragenic CpG islands (iCpGIs), which are crucial for transcriptional control and chromatin state modulation, were investigated. **Methods:** By performing a phylo-epigenetic comparison across 12 primate species, strong conservation of CpG-rich regions, punctuated by lineage-specific CpG transitions, particularly CpG→TpG and CpG→CpA was identified. **Results:** These mutational patterns align with methylation-dependent deamination mechanisms and highlight iCpGIs as evolutionarily constrained, epigenetically plastic elements. Notably, CpG variation alone recapitulated known primate phylogenies, suggesting that methylation-sensitive sites within iCpGIs encode both developmental and evolutionary information. **Conclusions:** It is proposed that such sites are prone to Environmentally Determined Epimutations (EDEMs)—methylation-driven, nutrition-sensitive changes that persist across generations and modulate gene regulatory capacity. This integrative framework advances Waddington’s concept of canalization by providing a molecular mechanism through which environmental factors can reshape developmental trajectories and contribute to evolutionary innovation.

## 1. Introduction

Conrad Hal Waddington envisioned evolution as a long-term process governing life. Across countless generations, the successful interaction of organisms with their environment has yielded increasingly robust life forms [1]. These organisms possess the intrinsic capacity to respond to fluctuating environmental conditions and, over successive generations, become progressively better adapted. Notably, certain acquired characteristics manifest already during embryonic development [1]. New individuals inherit the capacity not only to survive and reproduce, but also to pass on both ancestral and newly acquired traits to their offspring.

In Waddington’s view, a complete understanding of this adaptive progression requires expanding the Neo-Darwinian framework, which combines natural selection with random mutation, by incorporating a crucial epigenetic component [1]. This addition provides a missing link: a functional bridge between environmental pressures and the organism’s adaptive developmental responses.

Waddington’s work gave rise to two foundational concepts. First, he proposed that environmental stressors can induce phenotypic changes that eventually become genetically assimilated and heritable [1,2]. A central question that arises is how does such genetic assimilation manifest in the DNA itself?

Second, he introduced the concept of canalization, which describes the robustness of developmental processes [1,2]. It is evident that immediately following conception, the zygote already harbors all the essential information required to initiate an evolutionarily canalized developmental program. This enables the formation of a complex, highly adapted organism—be it an ape or a human—well adapted to anticipated environmental conditions.

This raises several fundamental questions: In what form is the information that canalizes development encoded within the zygote? How is it propagated across generations? And how do environmental stimuli influence its transgenerational plasticity?

Significant progress in epigenetics now allows us to assert with confidence that epigenetic regulation is as fundamentally important as classical genetics—a principle that holds true across biological disciplines, from cancer biology to embryology and evolutionary science. Integrating this expanding body of knowledge into evolutionary theory could deepen our understanding of processes that unfold on evolutionary time scales, which are otherwise only accessible through fragmentary, present-day observations.

It is well established that genome-wide, cell type-specific DNA methylation patterns demarcate active and repressed genomic regions and are central to establishing correct baseline gene expression profiles.

DNA methylation, which occurs predominantly at CpG dinucleotides in mammalian genomes, has been extensively studied over the past four decades and is widely recognized as a repressive epigenetic mark [3]. Over 60% of human genes possess a CpG-rich 5′ region, characterized by at least 50% of GC content [4], known as a CpG island (CGI), typically spanning 0.4–2 kb and surrounding the transcription start site of developmental, tissue-specific, and housekeeping genes [5]. Notably, the promoter CGIs of housekeeping genes remain consistently unmethylated throughout development and across all differentiated tissues, consistent with their ubiquitous expression [5]. This hypomethylated state serves to protect these CpG-rich regions from mutational erosion [6].

Spontaneous hydrolytic deamination of cytosine leads to uracil, which is efficiently detected and repaired [6]. In contrast, 5-methylcytosine deamination occurs 2- to 3-fold more frequently and generates TpG mismatches, which are less efficiently corrected [7]. Consequently, the transition rate from methylated CpG to TpG is 10–50 times higher than for other dinucleotide transitions [8,9]. Due to the palindromic nature of CpGs, this leads to CpG-to-TpG/CpA erosion over evolutionary time.

Studies analyzing more than 30 silenced and 100 non-silenced genes have shown that methylation of a relatively small core CGI region overlapping the transcription start site correlates consistently with gene silencing [10]. Depending on their precise methylation state, CpG sites within a CGI can have varying effects on gene expression, reflecting a complex and still not fully resolved regulatory logic [10].

Importantly, methylation extending into exonic regions does not necessarily block transcription initiation or elongation [10,11]. On the contrary, evidence indicates that methylation within gene bodies can modulate gene expression by enhancing transcription and affecting alternative splicing [12,13]. For instance, the CpG architecture at exon–intron junctions influences DNA methylation’s role in co-transcriptional splicing [13]. Another study found that exonic DNA methylation interacts with nucleosome occupancy and the histone mark H3K36me3 to regulate splicing outcomes [14].

Intragenic CpG islands (iCpGIs) have emerged as key regulatory elements, with their influence dependent on CpG architecture [13]. CpG density of CGIs is an important regulatory feature that contributes to the formation of histone signatures associated with transcription [15]. During development, unmethylated iCpGIs can adopt bivalent chromatin states, marked by both H3K27me3 (repressive) and H3K4me3 (activating) histone marks [16,17]. This poised configuration allows developmental genes to remain transcriptionally silent yet ready for rapid activation. As embryonic stem (ES) cells differentiate, these bivalent domains resolve into either active or repressive chromatin, depending on lineage commitment [17].

Moreover, intragenic CpG islands positioning within genes is not arbitrary and is instead selected for; iCpGIs can influence pre-mRNA processing, affecting mRNA isoform length and enhancer function, depending on their methylation status [16]. They contribute to the expansion of transcriptomic diversity. ICpGIs are frequently subject to DNA methylation changes during development, implying that their epigenetic regulation is crucial for tissue specific programming [16].

DNA methylation is thus a central determinant of mammalian embryogenesis. It is essential for embryonic viability [18], and its absence severely impairs differentiation [19]. Despite this, we are only beginning to uncover the full extent of its developmental impact [18].

Key pluripotency regulators such as *OCT4*, *SOX2*, and *hTERT* are under epigenetic control [20,21,22,23,24,25,26]. Their CpG islands spanning promoters and exons are unmethylated in early development and become subsequently methylated upon differentiation [27]. Furthermore, *SOX2* has been shown to be marked by bivalent histone modifications, and *hTERT* is subject to regulation via histone modifications and DNA methylation [25].

Following fertilization, global epigenetic reprogramming resets the methylation landscape, and essential pluripotency genes such as *Oct4* and *Sox2* become transcriptionally activated [28]. Together, these genes initiate a regulatory network involving hundreds to thousands of developmental genes. The precise expression balance between *Oct4*, *Sox2*, and their targets is critical for maintaining pluripotency, enabling the generation of all three embryonic germ layers [28]. Even minor deviations in expression levels of *Oct4* or *Sox2* can tip the balance toward differentiation, with long-term effects on embryonic development [28].

As differentiation begins, pluripotency-related transcriptional circuits are silenced, in part via de novo DNA methylation [19]. In this context, it has been observed that OCT4 fails to bind its target sites in embryonic stem cells (ESCs) when methylation occurs within 100 bp on each side of the target sequence [11].

Additionally, rapidly dividing ESCs must address telomere maintenance, as DNA polymerases cannot fully replicate chromosome ends [29]. Robust telomerase activity—regulated via transcriptional, epigenetic, and splicing mechanisms—ensures telomere length, self-renewal, and protection against premature differentiation [29,30,31,32,33,34].

In humans, telomerase has been detected in germ-line tissues, blastocysts, and 16–20-week-old fetal tissue, but not in most normal somatic tissues [35]. Telomerase expression can impact the expression of a variety of genes required for extended self-renewal and lifespan [36]. Undifferentiated stem cells are responsible for telomerase activity in the human fetus, and downregulation of telomerase activity occurs during differentiation and gestational development [35]. It has been demonstrated that an alternative splicing event, centered around *hTERT* exon 2, triggers *hTERT* mRNA decay in differentiating cells, whereas in pluripotent cells, inclusion of exon 2 promotes telomerase accumulation [34]. This tissue-specific and developmental regulation of telomerase in the human fetus suggests an important role for this ribonucleoprotein in human fetal tissue differentiation and development [35]. Thus, the regulation mechanisms finely tune the downregulation of telomerase right at the beginning of and during the various differentiation paths to be pursued, constituting a crucial adjusting screw for the developmental fate of the embryo.

Taken together, the activity of these three hierarchically positioned master genes and their target networks must be precisely tuned. Small perturbations in the epigenetic regulation of *OCT4*, *SOX2*, or *hTERT* can have profound consequences on the outcome of embryogenesis—ultimately affecting the development of a viable, well-adapted organism.

All three genes harbor intragenic CpG islands extending from upstream regions near the transcription start site into exon and intron (except *SOX2*) sequences: *OCT4* iCpGI spans exon 1 and intron 1; *SOX2* iCpGI includes the entire coding sequence of the single exon; and *hTERT* iCpGI covers exon 1, intron 1, exon 2, and part of intron 2.

Given the central importance of DNA methylation in early developmental regulation, it is hypothesized here that evolutionary changes in the CpG profiles, the basis of differential methylation of these crucial epigenetic regulatory regions, may represent a key feature of mammalian evolution.

To explore this, a phylo-epigenetic comparison of CpG versus non-CpG dinucleotide variation in the intragenic CpG islands (iCpGIs) of *OCT4*, *SOX2*, and *hTERT* across 12 primate species has been performed. These included hominins (*Homo sapiens*, *H. neanderthalensis*, and *Denisovan human*), great apes (*Pan troglodytes*, *Pan paniscus*, *Gorilla gorilla*, *Pongo abelii*), and Old World monkeys (*Macaca mulatta*, *Macaca fascicularis*, *Papio anubis*, *Chlorocebus sabaeus*, and *Rhinopithecus roxellana*).

Among extant species, the common chimpanzee and bonobo are the closest relatives to humans, having diverged ~1.7 million years ago [37]. The Neanderthal and Denisovan genomes were included as calibration points. Neanderthals and modern humans are estimated to have diverged at least 430,000 years ago [38,39], while Denisovans represent a distinct lineage that separated from modern humans approximately 600,000–800,000 years ago based on nuclear and mitochondrial DNA analyses [40,41,42].

## 2. Material and Methods

To investigate the evolutionary conservation and divergence of epigenetically relevant CpG-rich regions, three canonical pluripotency-associated genes—*OCT4* (*POU5F1*), *SOX2*, and *hTERT*—were selected based on their well-established regulation via DNA methylation. The reference human mRNA and genomic sequences of these genes were retrieved from the NCBI nucleotide database (https://www.ncbi.nlm.nih.gov/nuccore/?term=, accessed on 20 February 2025) under the following accession numbers: OCT4—NM_002701, BX088580; SOX2—NM_003106, AC117415; hTERT—NM_198253, NW_001838923.

The intragenic CpG islands (iCpGIs) within the 5′ regions of these genes were identified and extracted for twelve primate species using the UCSC Genome Browser (http://genome.ucsc.edu, accessed on 5 March 2025) [43]. These species included *P. troglodytes* (panTro6), *P. paniscus* (panPan3), *G. gorilla* (gorGor6), *P. abelii* (ponAbe3), *R. roxellana* (rhiRox1), *P. anubis* (papAnu4), *M. fascicularis* (macFas5), *C. sabaeus* (chlSab2), *M. mulatta* (rheMac10), archaic human relatives, *H. neanderthalensis* (Altai Neanderthal), and Denisova hominin, with sequences obtained from the Max Planck Institute’s JBrowse genome browser (https://bioinf.eva.mpg.de/jbrowse/, v1.12.1; accessed on 30 March 2025) [39].

To exclude potentially confounding repetitive elements (e.g., LINE-1, Alu), CpG-rich regions were screened using RepeatMasker (https://www.repeatmasker.org; accessed on 8 April 2025). The concatenated iCpGI sequences of *OCT4*, *SOX2*, and *hTERT* (in that order) were aligned using the multiple sequence alignment tool MAFFT (v7.511) with default settings and the nucleotide scoring matrix 1PAM/κ = 2, optimized for closely related sequences. The FFT-NS-i algorithm was employed to refine the alignment (https://mafft.cbrc.jp/alignment/server/; accessed on 15 April 2025) [44,45].

Phylogenetic relationships were inferred using the UPGMA clustering method based on the molecular clock assumption (https://mafft.cbrc.jp/alignment/server/phylogeny.html; accessed on 30 April 2025) [46]. To explore patterns of CpG conservation and divergence, all orthologous CpG dinucleotide positions were annotated and systematically converted: conserved CpGs across all species were replaced with “A”, while same-position dinucleotides lacking universal CpG conservation (i.e., variable CpG presence) were replaced with “T”. 

Visual inspection and comparison of phylogenetic trees were performed using Archaeopteryx.js (http://phylo.io; accessed on 30 April 2025) [47]. Pairwise sequence identity was assessed using Clustal 2.1 to generate a percent identity matrix (https://www.ebi.ac.uk/; accessed on 30 April 2025).

## 3. Results

**Figure 1:**
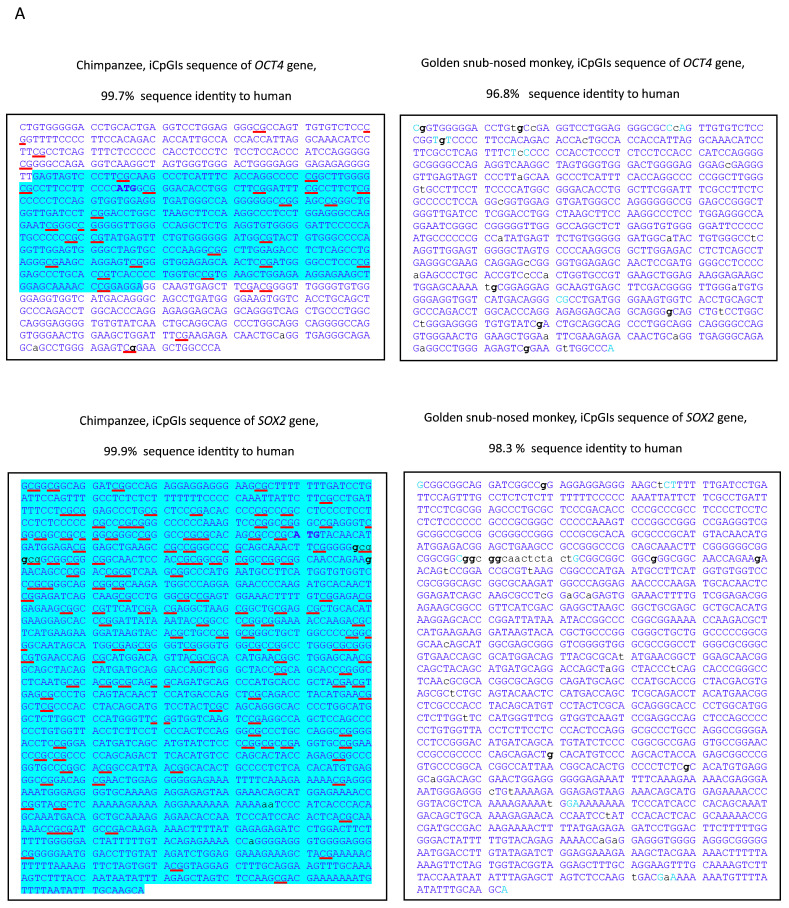

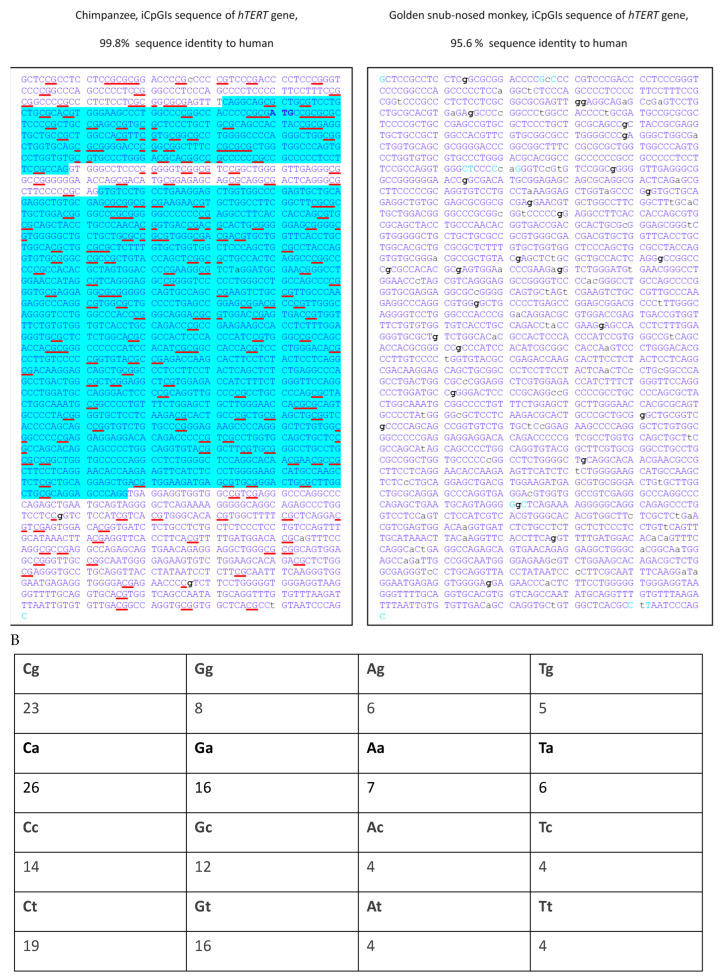
Comparative analysis of iCpGIs in *OCT4*, *SOX2*, and *hTERT* between human, chimpanzee, and golden snub-nosed monkey. (**A**) Representative excerpts from the UCSC Genome Browser BLAT alignment of the 5′ iCpGIs of *OCT4*, *SOX2*, and *hTERT* are shown for humans, chimpanzee, and golden snub-nosed monkey. Conserved nucleotides are denoted by uppercase blue letters; divergent bases appear in lowercase. Notably frequent X→G substitutions are highlighted in bold. CpG dinucleotides are underlined in red. The translation start codon is shown in bold blue uppercase. Exonic sequences are indicated by a light blue background. (**B**) The frequency of single-nucleotide substitutions involving guanine (“g”) versus non-“g” residues is quantified for both CpG and non-CpG contexts. The analysis includes all aligned iCpGI regions from *OCT4*, *SOX2*, and *hTERT* in chimpanzee and golden snub-nosed monkey. Gaps of ≥2 nucleotides were excluded from the analysis.

Initial sequence alignments were performed using the BLAT tool available via the UCSC Genome Browser, focusing on CpG-rich regions (iCpGIs) of the human genes *OCT4*, *SOX2*, and *hTERT*. These human sequences were first compared to their orthologs in the closely related chimpanzee (*P. troglodytes*) and subsequently to the more distantly related golden snub-nosed monkey (*R. roxellana*) (Figure 1A). All three iCpGIs exhibited dense clusters of CpG dinucleotides, spanning the first exon and extending into intron 1 in *OCT4*, and in the case of *hTERT*, further including exon 2 and part of intron 2.

These initial alignments revealed high sequence identity between human and chimpanzee: 99.7% for *OCT4*, 99.9% for *SOX2*, and 99.8% for *hTERT* (Figure 1A). Sequence identity between human and the golden snub-nosed monkey was moderately lower: 96.8% (*OCT4*), 98.3% (*SOX2*), and 95.6% (*hTERT*). Notably, the Colobinae subfamily, to which the golden snub-nosed monkey belongs, diverged from the Cercopithecinae (e.g., macaques, baboons, green monkeys) approximately 16.2 million years ago, as supported by fossil-calibrated molecular data [48]. In comparison, the divergence between orangutans and African apes occurred more recently, approximately 14 million years ago [48].

Across these alignments, nucleotide substitutions were frequently observed at guanine (G) positions. This trend is particularly evident in the *hTERT* iCpGIs of *R. roxellana*, where divergent G positions are marked in bold in the sequence alignment (Figure 1A). Further inspection of all interspecies alignments shown in Figure 1 revealed that these altered guanines were distributed across 23 CpG, 8 GpG, 6 ApG, and 5 TpG dinucleotides in the chimpanzee and golden snub-nosed monkey sequences. Additionally, adenine changes in 26 CpA dinucleotides were found (Figure 1B). All other changed dinucleotides appeared less frequently than the CpG and CpA dinucleotides (Figure 1B). Of note, none of the 18 CpG sites within the exonic regions of the *OCT4* and *SOX2* genes exhibiting interspecies variation between human, chimpanzee, and golden snub-nosed monkey resulted in an amino acid change.

These observations motivated the inclusion of additional primate species, which we used to perform a comprehensive alignment of the iCpGIs from *OCT4*, *SOX2*, and *hTERT*, resulting in a dataset comprising the introduced 12 primates. The aligned sequences ranged from 4928 to 5000 nucleotides, with an average length of 4977 nucleotides.

Within this full alignment, inspecting orthologous single-nucleotide positions, 132 single-nucleotide positions were changed outside of CpG dinucleotides in at least one of the primate sequences, corresponding to 2.7% of all nucleotide positions. Assuming these changes affect dinucleotide positions, 5.3% of all possible orthologous dinucleotides were altered. Of the 2489 total dinucleotide positions analyzed, 426 were orthologous CpG dinucleotide positions. Among these, 143 (34%) were variable in at least one species, translating to about 5.9% of all 2489 dinucleotides, while 283 (66%) were fully conserved. It should be noted that of the 143 orthologous CpG positions with variability between the selected species, 47 (33%) have either a CpG in all representatives of the hominid group or in the Old World monkey group. These differences at these orthologous CpG positions therefore distinguish the two major groups. There are 34 single-nucleotide variants (SNVs) at non-CpG positions that do the same. This translate to 25% of all orthologous SNV positions of all variant non-CpG positions.

**Figure 2:**
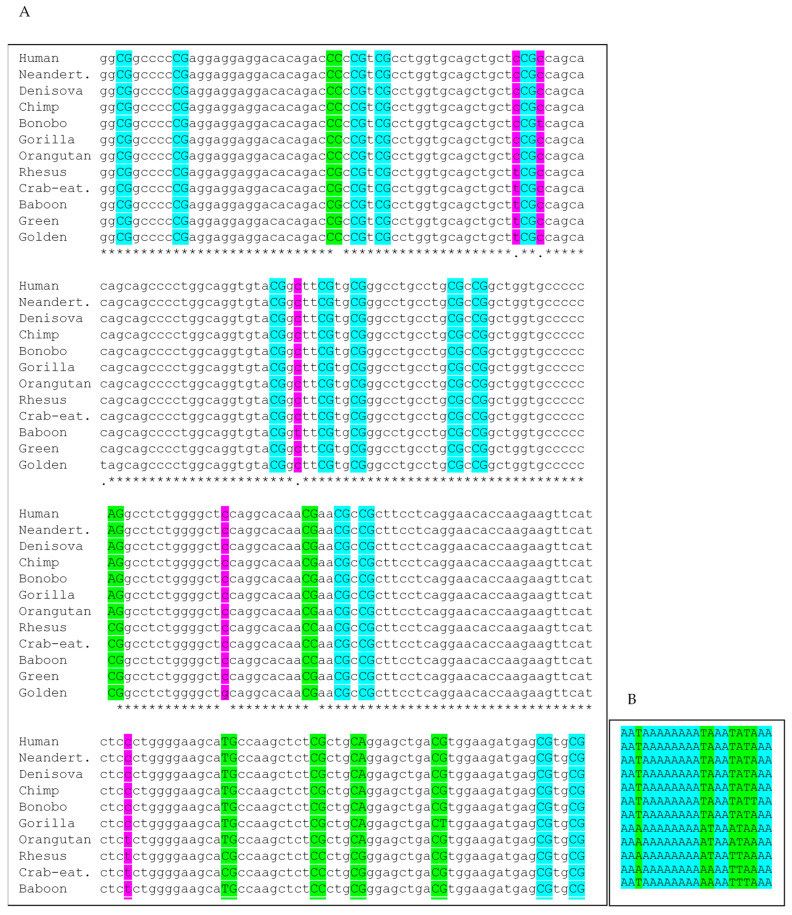

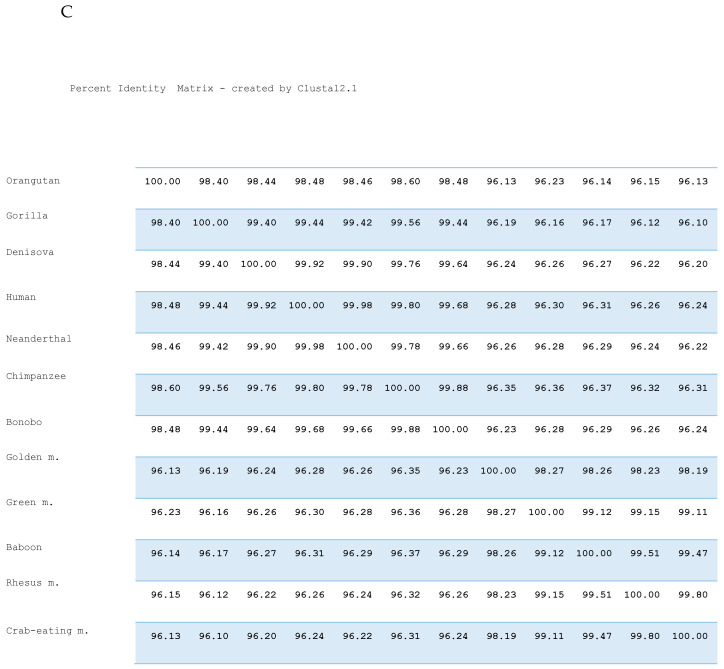
Conservation and divergence of iCpGIs across twelve primate species. (**A**) A segment (last part exon 2 *hTERT*) of a multiple alignment (~4977 nt average length) representing the concatenated iCpGI sequences of *OCT4*, *SOX2*, and *hTERT* from twelve primates. Purple highlights denote species-specific single-nucleotide variants (SNVs) in non-CpG contexts (5.3% of all aligned dinucleotide positions). Green marks variable CpG sites—positions where a CpG occurs in at least one species and is absent or mutated in others (5.9% of all positions, 34% of CpG sites). Universally conserved CpG sites are marked with light blue. Asterisk (*) indicate fully conserved nucleotides; single substitutions are marked with a dot. (**B**) Corresponding “A/T” transformation of the alignment: all CpGs are replaced with “A”, and all orthologous non-CpG dinucleotides at positions containing a CpG in at least one species are replaced by “T”. Continuous CpG blocks are shown with a light blue background, with interrupted (non-uniform) CpG positions in green. (**C**) A clustal 2.1-derived percent identity matrix depicting pairwise similarity across the twelve full-length sequences (~4977 nt each).

A representative 240-nucleotide alignment excerpt (Figure 2A) highlights conserved and altered CpG and non-CpG dinucleotide positions across species. Percent identity matrix analyses showed over 99% sequence identity among all hominid species except *P. abelii* (98%). Among the Old World monkeys, intra-group sequence identity was similarly high (≥99%), with *R. roxellana* again showing slightly reduced identity (98%). Inter-clade identity (Old World monkeys vs. hominids) averaged 96%, underscoring the high degree of conservation in these regulatory sequences.

These results contrast with prior studies of CpG islands in highly conserved housekeeping genes, where 4.8% of all orthologous dinucleotides were altered at non-CpG sites and 3.2% at orthologous CpG positions across human and great ape lineages [49]. In the present study, when focusing exclusively on the same hominins and great apes, 1.6% of non-CpG and 1.8% of CpG dinucleotides were variable, corresponding to 12% of all orthologous CpG sites—suggesting an even greater evolutionary constraint on iCpGIs associated with pluripotency genes.

Detailed analysis of CpG changes of the full alignment revealed specific substitution patterns: CG→CA (*n* = 45), CG→TG (*n* = 42), CG→CC (*n* = 23), CG→CT (*n* = 16), CG→GG (*n* = 15), and CG→AG (*n* = 20), with rarer transitions/transversions such as CG→AC, GC, GA, TA, and TT (*n* ≤ 3). CpA (on the sense strand) corresponds to TpG on the antisense strand, both of which are typical outcomes of methylated CpG deamination events. Of note, T→G belongs to the medium frequency substitutions in the eukaryotic genomes [50], suggesting that the slightly increased CG→CC rate could have resulted from the CG→TG transition and the subsequent TG→GG transversion in the complementary strand.

To visualize evolutionary dynamics specifically at CpG sites, a subalignment was generated by recoding conserved CpG dinucleotides as “A” and altered CpG positions as “T.” A representative excerpt is shown in Figure 2B. This transformation highlights the elevated substitution rate at CpG dinucleotides and the predominance of CG→CA and CG→TG transitions within these developmental regulatory regions.

**Figure 3:**
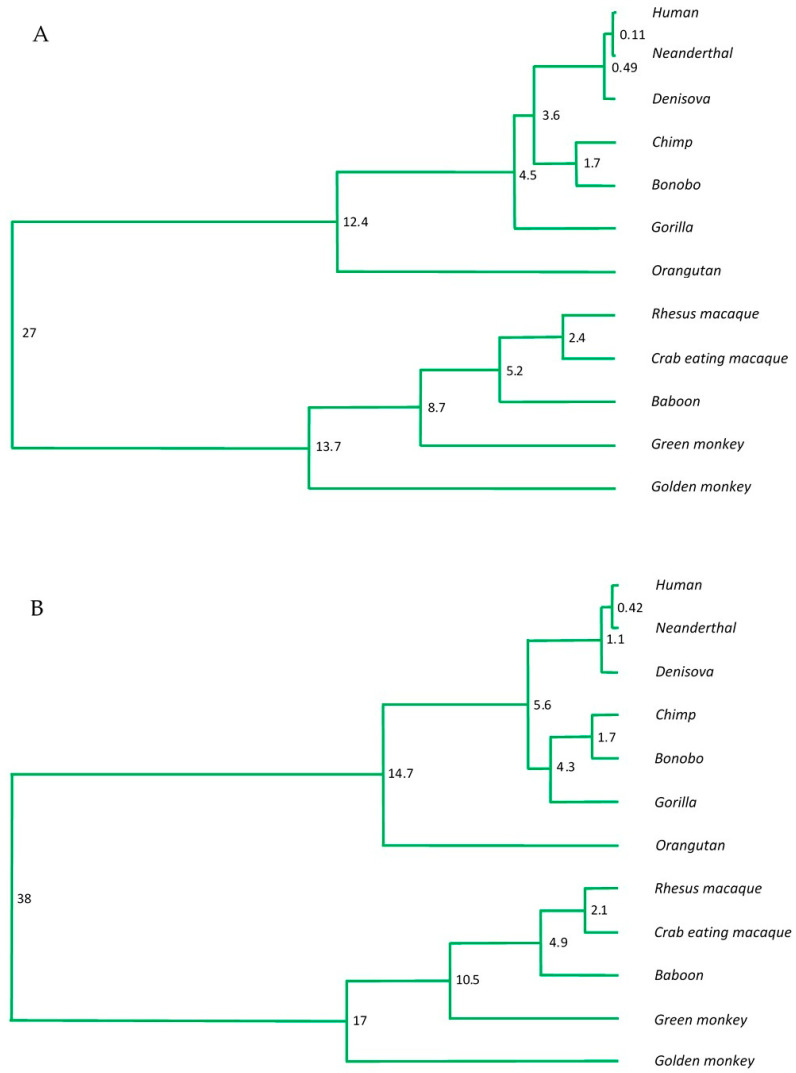
Phylogenetic and “phylo-epigenetic” relationships among 12 primate species based on *OCT4*, *SOX2*, and *hTERT* iCpGIs. (**A**) Standard phylogram based on all single-nucleotide polymorphisms (SNPs), including CpG and non-CpG substitutions, insertions, and deletions, reconstructed from aligned iCpGI sequences. (**B**) “Phylo-epigenetic” tree based exclusively on patterns of CpG conservation and divergence: conserved CpGs and all species-specific CpG substitutions within CpG islands of *OCT4*, *SOX2*, and *hTERT* were used to reconstruct alternative hierarchy reflecting epigenetic divergence. Numbers at branch nodes indicate divergence events, with values representing millions of years.

To further explore evolutionary relationships, two UPGMA phylogenetic trees were constructed: one based on all nucleotide changes (including CpG sites, insertions, and deletions) and another based solely on CpG dinucleotides and changes at orthologous positions harboring CpGs in at least one species (Figure 3A and Figure 3B, respectively). The latter, referred to as a “phylo-epigenetic tree”, more accurately recapitulated the evolutionary relationships and divergence times previously inferred from genome-wide analyses and fossil records [49].

Calibration of both trees was anchored to the divergence of *P. troglodytes* and *P. paniscus*, estimated at 1.7 million years ago [37]. The phylo-epigenetic tree reliably reconstructed established divergence events, including the ~14 Ma separation of orangutans from African apes, the ~16.2 Ma split between Colobinae and Cercopithecinae, and the ~6 Ma divergence between humans and chimpanzees, consistent with both fossil and molecular evidence [37,51,52,53,54,55].

Collectively, these findings demonstrate that CpG-rich regions of *OCT4*, *SOX2*, and *hTERT* exhibit strong evolutionary conservation across primates, with a subset of orthologous CpG sites undergoing lineage-specific transitions consistent with known epigenetic mutational mechanisms. These patterns underscore the relevance of iCpGIs as evolutionarily constrained elements involved in developmental gene regulation and suggest potential utility in phylo-epigenetic reconstructions.

## 4. Discussion

Fundamental questions concerning the nature and direction of embryonic and evolutionary development have long captured scientific interest. Pioneering work by Conrad Waddington introduced the concepts of developmental canalization and the genetic assimilation of acquired traits into heritable forms—cornerstones of epigenetic thought [1,2]. The present study aimed to extend Waddington’s framework by integrating over seven decades of subsequent advances in molecular biology and epigenetics, particularly as they relate to early embryonic development.

This work represents an attempt to further pursue the central postulates Waddington articulated, namely that the critical information guiding development must be encoded in the zygote and that this information must be modifiable to accommodate environmental input—both at the transgenerational and evolutionary scale.

To explore this hypothesis, interspecies sequence variation has been examined across a panel of primates, focusing on highly conserved intragenic CpG islands (iCpGIs) within key pluripotency-associated genes. The analysis revealed that the most frequently observed genetic changes involved CpG dinucleotides, with a notable predominance of CpG-to-TpG and CpA transitions. Moreover 33% of the variable CpG positions were uniformly altered either in the hominid or the Old World clade, hence forming a clear distinguishing mark (Figure 3).

While this finding aligns with known molecular mechanisms—specifically, the elevated deamination rate of 5-methylcytosine at CpG sites—it holds further significance in light of recent observations. CpG-to-TpG mutations have been shown to frequently generate de novo binding sites for transcription factors such as OCT4 with higher functional efficiency than other mutational events, thereby increasing regulatory plasticity and potentially conferring an evolutionary advantage [56]. Similar perspectives underscore that these transitions contribute to genomic flexibility and can be associated with phenotypic innovations relevant to speciation [57].

What is more surprising, however, is the specific localization of these frequently occurring CpG changes within the iCpGIs of master developmental regulators. It is well established that CpG density and methylation status within such regions play critical roles in modulating gene expression. Importantly, *OCT4*, *SOX2*, and *hTERT* are situated at the apex of the regulatory hierarchy that orchestrates pluripotency and lineage specification. Even minor perturbations in the expression or epigenetic regulation of these genes are known to propagate through downstream networks, altering the tightly coordinated spatiotemporal gene expression patterns required for proper morphogenesis and differentiation [58].

Environmental factors—most prominently, nutrition—are known to shape the DNA methylation landscape on a genome-wide scale [59]. Approximately 28 million CpG dinucleotides and their methylation pattern in the human genome must be faithfully maintained during somatic cell division to preserve phenotypic identity [60]. S-adenosylmethionine (SAM), derived from dietary components such as folate, methionine, and riboflavin, serves as the principal methyl donor in one-carbon metabolism and DNA methylation processes [59]. Epigenetically metastable genomic regions are particularly sensitive to fluctuations in SAM availability and nutritional input during early embryogenesis, and several studies have confirmed that maternal diet can influence heritable epigenetic phenotypes in offspring [61,62].

Importantly, it has been demonstrated that CpG islands, although typically protected from methylation, may acquire aberrant methylation patterns following transient local sequence alterations. Such changes can escape erasure and be transmitted across generations [63]. Mutations that impact epigenetic stability and expression variability have thus been proposed to offer selective advantages in fluctuating environments [63,64].

It has been further hypothesized that during the critical epigenetic reprogramming window of the zygote and early embryo, incomplete demethylation events may allow residual methylation marks to persist at specific CpG sites—particularly within iCpGIs of developmental genes. These sites may act as epigenetically metastable loci whose modulation could influence gene dosage and, ultimately, phenotype [65].

Given that complete demethylation in primordial germ cells has been experimentally confirmed [64], it is proposed here that isolated CpG methylation signatures—particularly those within iCpGIs of pluripotency genes—may occasionally escape this process. Their persistence, although infrequent, may underlie subtle heritable changes that only become evident across evolutionary timescales. Moreover, the original methylation signal may be masked by subsequent deamination-induced transitions (e.g., CpG to TpG), obscuring their historical epigenetic origin.

Remarkably, phylogenetic trees constructed solely on CpG variation within these conserved gene regions (Figure 3B) recapitulate species relationships consistent with phylogenies derived from whole-genome and mitochondrial DNA analyses, as well as fossil records. This finding highlights the evolutionary informativeness of CpG variation and supports the notion that epigenetically mediated sequence changes contribute to lineage divergence.

It is therefore proposed that CpG transitions within regulatory regions of developmental genes represent a distinct class of mutations: Environmentally Determined Epimutations (EDEMs). These do not conform to the classical concept of random mutations central to the modern synthesis but rather arise via methylation-dependent processes modulated by environmental factors such as diet. Over evolutionary time, these CpG alterations may exert profound effects on embryonic development by reshaping the regulatory capacity of master genes.

This leads to the important inference that artificial alteration of key CpGs—e.g., by CRISPR/Cas-based CpG editing—could significantly impact embryonic development. Suggested first test candidates are those CpGs distinguishing hominids and New World monkeys.

Such CpGs may constitute pivotal epigenetic control points, and their modification may yield unpredictable and potentially detrimental consequences. Provocatively speaking, they may contribute to fundamental differences between humans and apes.

Taken together, these findings close the conceptual loop connecting environmental inputs, epigenetic regulation, developmental processes, and evolutionary trajectories. They provide a molecular framework that supports Waddington’s postulate of acquired character assimilation and offer a mechanistic explanation for how developmental pathways may canalize and branch in response to persistent environmental pressures—most notably, food availability.

It is proposed here that in an evolutionary timescale, the epigenetic regulation of the pluripotency network is influenced by the availability of food. This bridge between a crucial environmental factor for life and the epigenetic regulation of developmental genes is a flexible driver of evolution that enables adaptable rates of development. It is a possible answer to many inconsistencies in evolutionary biology that are related to the fact that various examples contradict gradualism [66], with the observation of unexpected rates of development.

## 5. Conclusions

This study provides first-time evidence that specific CpG sites within intragenic CpG islands of master developmental regulators serve as epigenetically metastable loci. They may act as molecular fulcrums that modulate the tempo of evolution in response to external environmental cues—most notably, nutritional input. The identification of these sites as hotspots for methylation-sensitive sequence transitions, coupled with their localization in key pluripotency genes, points to their dual role as both mediators and recorders of environmentally driven epigenetic change. These findings support the concept of Environmentally Determined Epimutations (EDEMs) as a distinct category of mutation whose origin is rooted in dynamic methylation processes rather than stochastic genomic alterations. Nutrition-sensitive methylation patterns at specific CpGs may shape evolutionary trajectories through modulation of gene regulation and expression plasticity. This work hints to an understanding of how epigenetic mechanisms may integrate environmental information into the genetic fabric of developmental programs. It thus offers a mechanistic bridge between fluctuating ecological contexts and variable evolutionary rates—providing a compelling alternative to purely gradualist models of biological change.

## Data Availability

All genomic sequences analyzed in this study are publicly available through the UCSC Genome Browser and Ensembl Genome Browser. Alignment data and annotated regions are available from the author upon request.
